# Supplementary material for: PIK3CAH1047R-induced paradoxical ERK activation results in resistance to BRAFV600E specific inhibitors in BRAFV600E PIK3CAH1047R double mutant thyroid tumors
Source: Oncotarget. 2017 Oct 11;8(61):103207–22. doi: 10.18632/oncotarget.21732 (PMC5732722; doi:10.18632/oncotarget.21732)
Supplement: Supplementary file 1 [file oncotarget-08-103207-s001.pdf]

PIK3CA^{H1047R}-induced paradoxical ERK activation results in resistance to BRAF^{V600E} specific inhibitors in BRAF^{V600E} PIK3CA^{H1047R} double mutant thyroid tumors

SUPPLEMENTARY MATERIALS

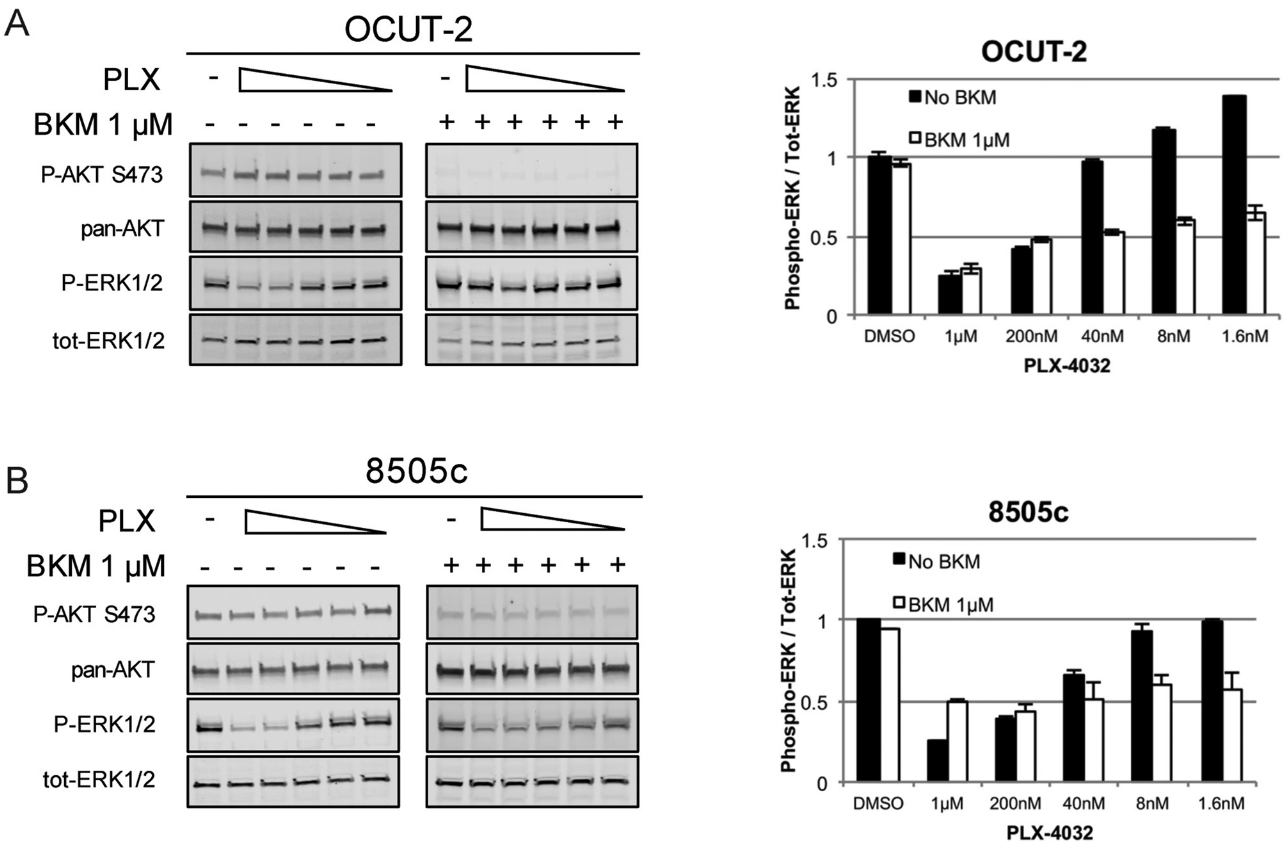

Supplementary Figure 1: Paradoxical activation of ERK abrogated by BKM-120. Representative western blot from total protein extracted from OCUT-2 (**A**) and 8505c (**B**) cells exposed to decreasing concentrations of PLX-4032 (from 1 µM to 1.6 nM) in presence or absence of BKM-120 at 1 µM. All experiments were performed in triplicates. The quantifications are expressed as average ratios of the three independent experiments.
